# Quantization Framework for Fast Spiking Neural Networks

**DOI:** 10.3389/fnins.2022.918793

**Published:** 2022-07-19

**Authors:** Chen Li, Lei Ma, Steve Furber

**Affiliations:** ^1^Advanced Processor Technologies (APT) Group, Department of Computer Science, The University of Manchester, Manchester, United Kingdom; ^2^Beijing Academy of Artificial Intelligence, Beijing, China; ^3^National Biomedical Imaging Center, Peking University, Beijing, China

**Keywords:** spiking neural networks, fast spiking neural networks, ANN-to-SNN conversion, inference latency, quantization, occasional noise

## Abstract

Compared with artificial neural networks (ANNs), spiking neural networks (SNNs) offer additional temporal dynamics with the compromise of lower information transmission rates through the use of spikes. When using an ANN-to-SNN conversion technique there is a direct link between the activation bit precision of the artificial neurons and the time required by the spiking neurons to represent the same bit precision. This implicit link suggests that techniques used to reduce the activation bit precision of ANNs, such as quantization, can help shorten the inference latency of SNNs. However, carrying ANN quantization knowledge over to SNNs is not straightforward, as there are many fundamental differences between them. Here we propose a quantization framework for fast SNNs (QFFS) to overcome these difficulties, providing a method to build SNNs with enhanced latency and reduced loss of accuracy relative to the baseline ANN model. In this framework, we promote the compatibility of ANN information quantization techniques with SNNs, and suppress “occasional noise” to minimize accuracy loss. The resulting SNNs overcome the accuracy degeneration observed previously in SNNs with a limited number of time steps and achieve an accuracy of 70.18% on ImageNet within 8 time steps. This is the first demonstration that SNNs built by ANN-to-SNN conversion can achieve a similar latency to SNNs built by direct training.

## 1. Introduction

Deep spiking neural networks (SNNs) use the revolutionary techniques developed for deep learning while retaining biological fidelity, with the objective of achieving power-efficient, low-latency, and high-performance computing. Their performance, and specifically their inference accuracy, has improved significantly over recent years, driven by the motivation to prove that SNNs are as functional as their artificial neural network (ANN) counterparts. Emerging techniques show that lossless SNN accuracy is possible (Diehl et al., 2015; Rueckauer et al., 2017; Sengupta et al., 2019; Deng and Gu, 2021; Li et al., 2021c).

With this success in the pursuit of inference accuracy, considerable scholarly attention has shifted to the aspect of inference latency, referred to as fast SNN research in this paper (Ho and Chang, 2020; Deng and Gu, 2021; Hwang et al., 2021; Li et al., 2021a). Fast SNNs are achieved either by conducting more efficient ANN-to-SNN conversion or by training the SNNs directly. Nevertheless, with the reduction in latency comes degradation in accuracy, resulting in the well-known accuracy-latency trade-off in SNNs.

Here we aim to build fast SNNs while avoiding accuracy loss. In particular, we choose an ANN-to-SNN conversion technique to minimize accuracy loss while applying a novel quantization framework to push latency below 10 time steps, for the first time. Thus, we demonstrate a highly effective method to build state-of-the-art ultra-fast, high-accuracy SNNs.

The key contributions of this paper are listed below.

- **Performance**: We overcome the accuracy loss problems previously seen in fast SNNs after conversion from ANNs, and achieve a state-of-the-art accuracy and latency. Specifically, on ImageNet we achieve the accuracy of 70.18% in 8 time steps and 74.36% in 10 times steps.
- **Information compression**: The fast SNNs are generated by compressing activation precision. We discuss how to achieve extremely low-bit activation compression (down to 2 bits) and, more importantly, how to ensure the compatibility of this technique with SNNs.
- **Noise suppression**: We identify a new type of noise in spiking neurons, which we call “occasional noise,” and show that it is the main obstacle to achieving competitive accuracy for fast SNNs. An effective approach is proposed to suppress its negative effect on SNN performance.
- **Framework**: A comprehensive quantization framework for fast SNNs (QFFS) is built including the proposed information compression, noise suppression techniques, and other techniques. This framework enables SNNs to be built with both high inference accuracy and low inference latency. Beyond that, further improvements in accuracy, latency, and biological plausibility are possible based on this framework.

## 2. Related Work

The inference latency of SNNs has continued to reduce over the last 5 years (Rueckauer et al., 2017; Sengupta et al., 2019; Ho and Chang, 2020; Lu and Sengupta, 2020; Deng and Gu, 2021; Hwang et al., 2021). The early demonstration of SNNs on ImageNet needed about 2,000 ms to get competitive accuracy (Sengupta et al., 2019). Rueckauer et al. (2017) applied a modified integrate-and-fire (IF) model and analog input to facilitate the accuracy and latency of SNNs. The 0.1–1% outliers were discarded to further reduce the SNN latency.

The modified IF model and analog input were then widely used in SNN research, and there was a surge of interest in further shortening the inference latency of the SNNs. Hwang et al. (2021) and Deng and Gu (2021) used a pre-charged membrane potential and bias shift, respectively to eliminate the systematic error during ANN-to-SNN conversion. Another contribution of Deng and Gu (2021) is they used clipped ReLU during ANN training to match the response curve of SNNs better. Ho and Chang (2020) applied clipped ReLU during ANN training as well, but the clipping point in each layer is trainable. Through these efforts, ANN-to-SNN conversion has become increasingly effective, and the inference latency of the SNNs has reduced to about 30 time steps. However, the accuracy loss after conversion is considerable when the latency is pushed down toward 10 time steps.

Another parallel method to achieve fast SNNs is to train the SNNs directly by surrogate gradients. Using this method, the inference latency can be pushed to several time steps. Nevertheless, direct training is hindered by the huge memory budget during training and the accuracy degeneration during inference (Fang et al., 2021).

The research into ANN quantization is extraordinarily prosperous, pursuing low computation and memory budgets for deploying Tiny Machine Learning applications on edge devices (Warden and Situnayake, 2019). The standard methods are post-training quantization and quantization-aware training (Krishnamoorthi, 2018). Using these two approaches, most neural network models can achieve lossless accuracy with 8-bit precision compared with the corresponding full precision models. Further reduction of the precision mainly relies on modifying gradients (Esser et al., 2019). The extreme situation is using 1-bit weights and activations to conduct inference, an approach called binary neural networks (BNNs) (Qin et al., 2020).

Research into applying quantization to SNNs is comparably limited. Quantization techniques are primarily adopted to compress the model footprint of the SNNs, and these techniques have been applied to weights, neuronal parameters, and neuronal state to deploy SNN algorithms on memory-constrained neuromorphic hardware (Schaefer and Joshi, 2020; Chowdhury et al., 2021; Lui and Neftci, 2021).

Meanwhile, several studies have explored the effectiveness of using quantization techniques to promote fast SNNs (Bu et al., 2021; Mueller et al., 2021; Wu et al., 2020). However, these methods either fail to scale to ImageNet, or suffer severe accuracy degradation. The main challenge for fast SNNs—preventing accuracy drop when pushing down the inference latency—has not been dealt with. The main differences between our research and these studies are:

- We conduct a comprehensive analysis of occasional noise and provide a corresponding noise suppression method, which is shown to be crucial to achieving competitive accuracy for SNNs within strictly limited time steps.
- Our research enables building SNNs with 2-bit precision and loss-less accuracy (while other research uses 4-bit to 8-bit quantization and suffers serious accuracy loss). Also, some key modifications to the standard quantization techniques are emphasized in this paper to better fit the dynamics of spiking neurons.
- Other methods usually apply analog neurons instead of spiking neurons in the output layer of the SNNs, to improve the resolution of the output layer so keeping competitive accuracy on tasks such as ImageNet. Our method shows the possibility of achieving an accuracy higher than 70% on ImageNet with spiking neurons in the output layer.

## 3. Motivation

The motivation for this research is to develop a practical method to reconcile the accuracy-latency trade-off in SNNs. Currently, there are two dominant methods to build SNNs: ANN-to-SNN conversion, and direct training with surrogate gradients. Which method is chosen depends on the requirements of the SNNs under different application scenarios. Generally, ANN-to-SNN conversion features high accuracy, while direct training features low latency. In other words, an accuracy-latency trade-off arises with current SNNs.

**Figure 1:**
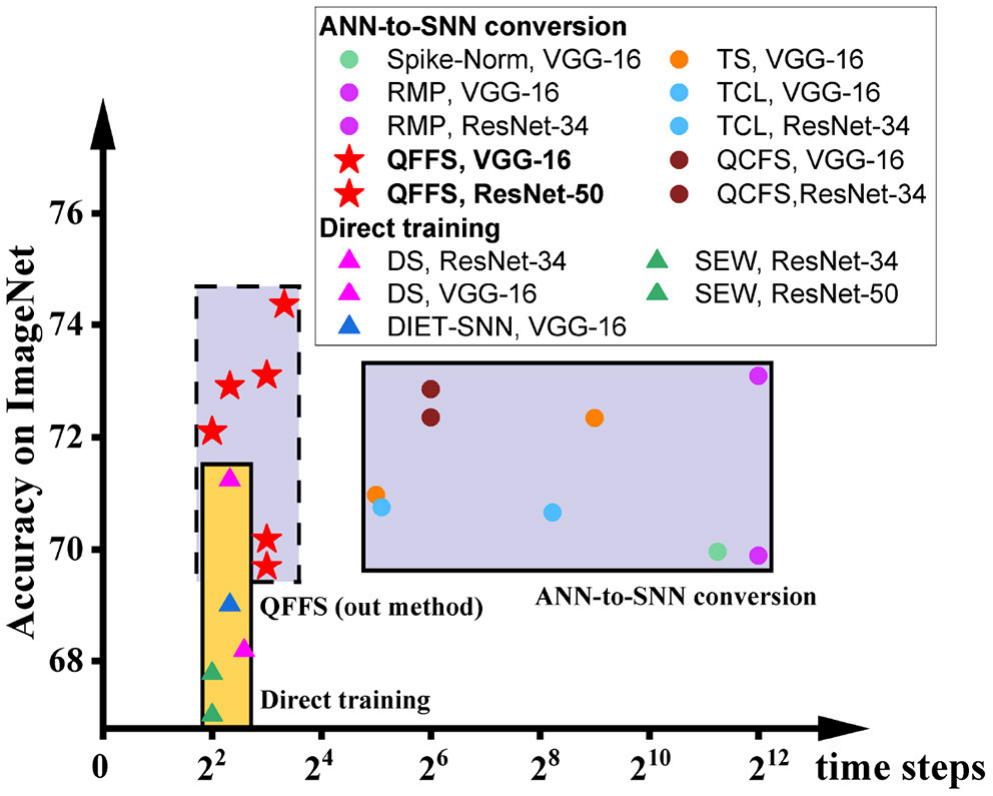
The accuracy and latency of SNNs built by different methods: Spike-Norm (Sengupta et al., 2019), TS (Deng and Gu, 2021), RMP (Han et al., 2020), TCL (Ho and Chang, 2020), QCFS (Bu et al., 2021) DS (Li et al., 2021b), SEW (Fang et al., 2021), DIET-SNN (Rathi and Roy, 2020), and QFFS proposed in this paper. ANN-to-SNN conversion delivers high accuracy, and direct training delivers low latency. The proposed QFFS approach pushes the latency, when using ANN-to-SNN conversion, to a level similar to that using direct training. Also, our method shows about 2.5% higher accuracy than the best accuracy achieved by direct training.

To illustrate this, we show the accuracy and latency of SNNs using these two methods in Figure 1, grouped by different symbols. It is obvious that these two methods are currently distinguished from each other and have distinct working zones. To date, there is no approach to building SNNs with latency matching that using direct training and accuracy equivalent to that using ANN-to-SNN conversion. The purpose of this paper is to push the bounds of both of these methods, to promote the reconciliation of the accuracy-latency trade-off in SNNs. Specifically, we focus on improving the latency of ANN-to-SNN conversion, for the first time to a level close to that of direct training. As a result, the latency gap between these two methods can be closed, while ANN-to-SNN conversion will show clear advantages in training (lower memory budget and shorter training time) and in inference (higher accuracy) than direct training.

## 4. Materials and Methods

### 4.1. Method Overview

Considering a rate-coded SNN built using the ANN-to-SNN conversion technique (Diehl et al., 2015; Rueckauer et al., 2017), the accuracy of the SNN, *Acc*(*SNN*), is given by


(1) $$\begin{array}{l} Acc\left(SNN\right)=Acc\left(ANN\right)-Loss\left(conversion\right) \end{array}$$

where *Acc*(*ANN*) is the accuracy of the full precision ANN, *Acc*(*SNN*) is the accuracy of the SNN, and how close this is to the full ANN accuracy depends on *Loss*(*conversion*), which is the accuracy loss introduced by ANN-to-SNN conversion. If we quantize the ANN prior to conversion then this becomes


(2) $$\begin{array}{l} Acc\left(SNN\right)=Acc\left(QuantANN\right)-Loss\left(conversion\right) \end{array}$$

where *Acc*(*QuantANN*) is the accuracy of the quantized ANN. After this modification, the target accuracy of the SNN becomes *Acc*(*QuantANN*). Benefitting from the recent advances in ANN quantization techniques, *Acc*(*QuantANN*) is increasingly close to *Acc*(*ANN*) even when the bit precision is strictly constrained. Hence, the baseline accuracy of the SNN, *Acc*(*QuantANN*), is maintained in principle.

Minimizing the ANN-to-SNN conversion loss *Loss*(*conversion*) is crucial to ensuring that the SNN approaches this baseline accuracy. We minimize the loss by analyzing the neuronal dynamics of spiking neurons to find the origin of the accuracy loss and eliminate it (Sections 4.3, 4.4).

As for the inference latency, we empirically show that the inference latency of the SNN and the activation bit-width of the ANN are correlated after ANN-to-SNN conversion, so a fast SNN can be built by using a quantized ANN. This is covered in the following section, where we describe the applied ANN quantization techniques and highlight the modifications to the standard quantization techniques to ensure compatibility with SNNs.

Another issue we address in this paper is the simulation of max pooling in SNNs. We propose a practical SNN max pooling method to improve the accuracy of the SNN compared with that using average pooling, without compromising its event-based nature.

**Figure 2:**
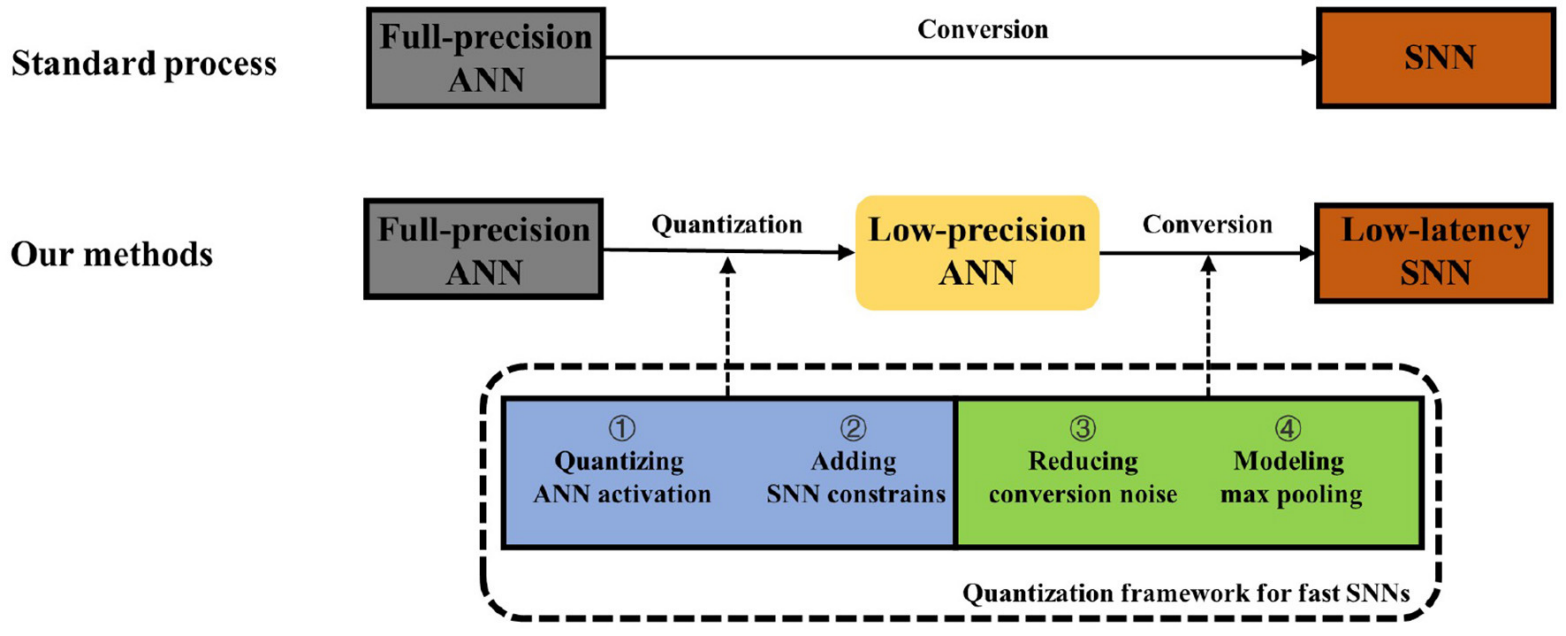
The general ANN-to-SNN conversion diagram and the approach we propose to achieve fast SNNs.

The exploration of these aspects forms a quantization framework for fast SNNs (QFFS) as shown in Figure 2. How this framework delivers further improvements in SNN performance is discussed at the end of the paper. The detailed equations related to the ANN-to-SNN conversion and Quant-ANN-to-SNN conversion are provided in Section 4.6.

### 4.2. Information Compression During Training

#### 4.2.1. Implementing Quantization Training

The inference accuracy of SNNs increases with the simulation time step but, in essence, it increases with the amount of information transmitted by uniform spike trains. After sufficient information has been accumulated, a comparatively reliable “decision” can be made, and this point in time is defined as the inference latency of the SNN. For example, to transmit 8-bit information, at least 255 ms is required in a rate-coded SNN with a 1 ms time resolution. If temporal coding is used, the required length of time is also related to the target information bit-width. Thus, reducing the required information bit-width is the key to achieving fast SNNs.

When using an ANN-to-SNN conversion technique, the required bit precision of the SNN is determined by the activation bit precision of the ANN. So the problem becomes that of building a quantized ANN with an activation bit-width as low as possible while maintaining high accuracy.

During the last decade, ANN quantization techniques have been at the center of much attention, and the standard quantization methods (post-training quantization and quantization-aware training) have increasingly matured. For instance, there are well-developed and easily accessed APIs in PyTorch to conduct ANN quantization by these two methods. Though these APIs are easy to access, these two standard methods are not suitable for this research, as they fail to achieve competitive accuracy in extremely low bit precision such as 2 bits. Hence, the first obstacle is to choose a more effective quantization method than the standard post-training quantization and quantization-aware training. This obstacle is also part of the reasons why early attempts to use ANN quantization to promote fast SNNs either failed to scale to challenging datasets (such as ImageNet) or suffered high accuracy loss in fast SNNs (e.g., 6% on ImageNet).

The ANN quantization technique chosen in this paper is based on LSQ (Esser et al., 2019). LSQ defines the gradients of the quantization step size to prevent activations from being too close to quantization transmission points. It can enable network quantization down to 2 bits while minimizing the accuracy loss introduced by quantization. This quantization accuracy loss equals *Acc*(*ANN*)−*Acc*(*QuantANN*), which is the accuracy difference between the full-precision ANN model and the quantized-ANN model. According to the original LSQ paper, this accuracy loss is about 3.2% for 2-bit ANN and 1.1% for 3-bit ANN on ResNet-50. How to reduce this accuracy loss is discussed in Section 6. Additionally, LSQ is not open-sourced, and our implementation has not achieved a similar accuracy to that claimed in that paper. For example, our 2-bit and 3-bit ANN quantization results on ImageNet are 1.5% and 2.3% lower than the reported results in the LSQ paper, respectively. This suggests that there is further scope for improving our methods.

#### 4.2.2. Modifications to Promote Compatibility With SNNs

As the quantized ANN will be converted into an SNN in the future, the ANN quantization technique should be compatible with the properties of SNNs.

We list the modifications to the general ANN quantization technique in Table 1. We only apply activation quantization during training and leave the input, weights, and biases as floating-point. In the standard quantization procedure, the model generated by fake quantization training will be converted to an integer model and run on different backends. Here we only apply the fake quantization training and then convert the model to an SNN. The granularity of activation quantization is the tensor. There are two modifications we found crucial to the final SNN performance:

**Table 1:** The main differences between general quantization techniques and the quantization techniques for fast SNNs.

	**Standard quantization**	**Quantization for fast SNNs**
Position	Network input, network output, ReLU, arithmetic	ReLU
Procedure	Fake quantization training-convert to integer model	Fake quantization training
	- run on the backend with integer arithmetic accelerator	
Operation	Rounding	Grounding
Granularity	Per tensor, per channel	Per tensor
Benefits	Speed up inference and reduce memory budget	Reduce inference latency

Firstly, many quantization techniques including the applied LSQ leave the output layer in floating-point to render better accuracy, e.g., 4% higher on ImageNet than that quantizing the output layer. However, modeling floating-point with spiking neurons is expensive. It needs either many time steps to generate enough spikes to reach the same precision, or to use integrate-but-not-fire neurons in the SNN and represent the high-precision information by the neurons' membrane potentials. These two solutions will damage the inference latency or biological plausibility of the built SNNs. In this research, we explore the sensitivity of SNN performance to the activation precision in the output layer, and choose an optimal precision to promote competitive accuracy and latency.

Secondly, the integrate-and-fire mechanism in spiking neurons corresponds to rounding down rather than rounding to nearest which is generally used in ANN quantization. To address this issue, we can either change to use the rounding down during the quantization training, or stick to using rounding to nearest during quantization and compensate for it later. Considering that the quantization method is fine-tuning an already trained full-precision model, rounding down during quantization will introduce a systematic error resulting in a considerable accuracy loss, especially for low-precision quantization. Here we stick to using rounding to nearest during quantization and compensate for it in the SNN by pre-charging the membrane potential (Hwang et al., 2021).

### 4.3. Occasional Noise

The types of noise causing accuracy loss during ANN-to-SNN conversion are summarized for the first time in Diehl et al. (2015), where three kinds of noise—sub-threshold noise, supra-threshold noise, and rate-coding noise—are illustrated. Some research categorizes these as errors rather than noise. Here, we stick to calling them noise, to emphasize their randomness and uncertainty.

Here we argue that there is a fourth kind of noise, which we call occasional noise. Occasional noise refers to the phenomenon that occasional spikes are generated in spiking neurons where they should not be. For example, consider an artificial neuron with an input of 0.4, and the threshold of the corresponding spiking neuron is 1 with a simulation period of 10 time steps. During this simulation period, the average input value is 0.4 and the neuron should generate 4 spikes in 10 time steps, in the simplest situation. However, the inputs of a spiking neuron are weighted spikes, with random timing. Possible situations include:

- The input of this spiking neuron is –1 in the first 9 time steps and 13 in the last time step. Here the number of spikes generated is 1 instead of 4.
- The input is 1 in the first 8 time steps and –2 in the last 2 time steps. Then the summed input is 8*1 + 2*(–2) = 4, which is correct, but the generated spike count is 8 instead of 4.

**Figure 3:**
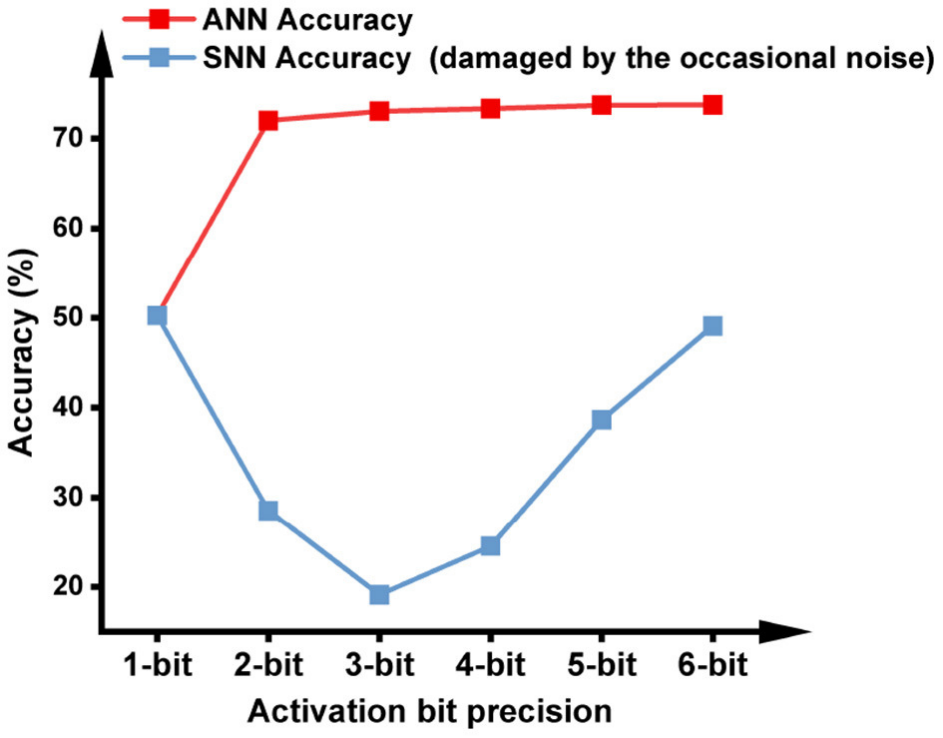
ANN accuracy after quantization training with different bit precisions, and SNN accuracy without handling the occasional noise. The model is VGG-16 and the dataset is ImageNet. The ANN-to-SNN conversion is based on the approach proposed in this paper to facilitate the conversion of low-bit activations.

These erroneously generated spikes will propagate through the network and cause an accuracy drop in the SNN. To verify this, We trained ANNs with different activation quantization precision, and evaluated their accuracy after converting to SNNs. As shown in Figure 3, the SNN accuracy is far from the baseline ANN accuracy for all activation precisions higher than 1 bit. Note that a 1-bit SNN can complete inference in one time step, then its function is equivalent to a 1-bit ANN so its conversion accuracy loss is zero. However, no temporal information was utilized during its inference, so the network is no longer spiking and is out of the scope of fast SNNs. The following section will discuss how this noise may be suppressed to achieve lossless Quant-ANN-to-SNN conversion.

### 4.4. Handling Occasional Noise and the Other Three Noise Types

To achieve lossless Quant-ANN-to-SNN conversion, occasional noise and the other three types of noise need to be handled. We will briefly introduce how to cope with the other three types of noise in the following section, as these three noise types have been researched for years. Then we will focus on illustrating the proposed approach to handle occasional noise.

#### 4.4.1. Handling the First Three Noise Types

By using analog inputs and the modified IF neuron, rate-coding noise is eliminated, and the dropped supra-threshold signal is recovered by the reset-by-subtraction mechanism in the modified IF model (Rueckauer et al., 2017).

**Figure 4:**
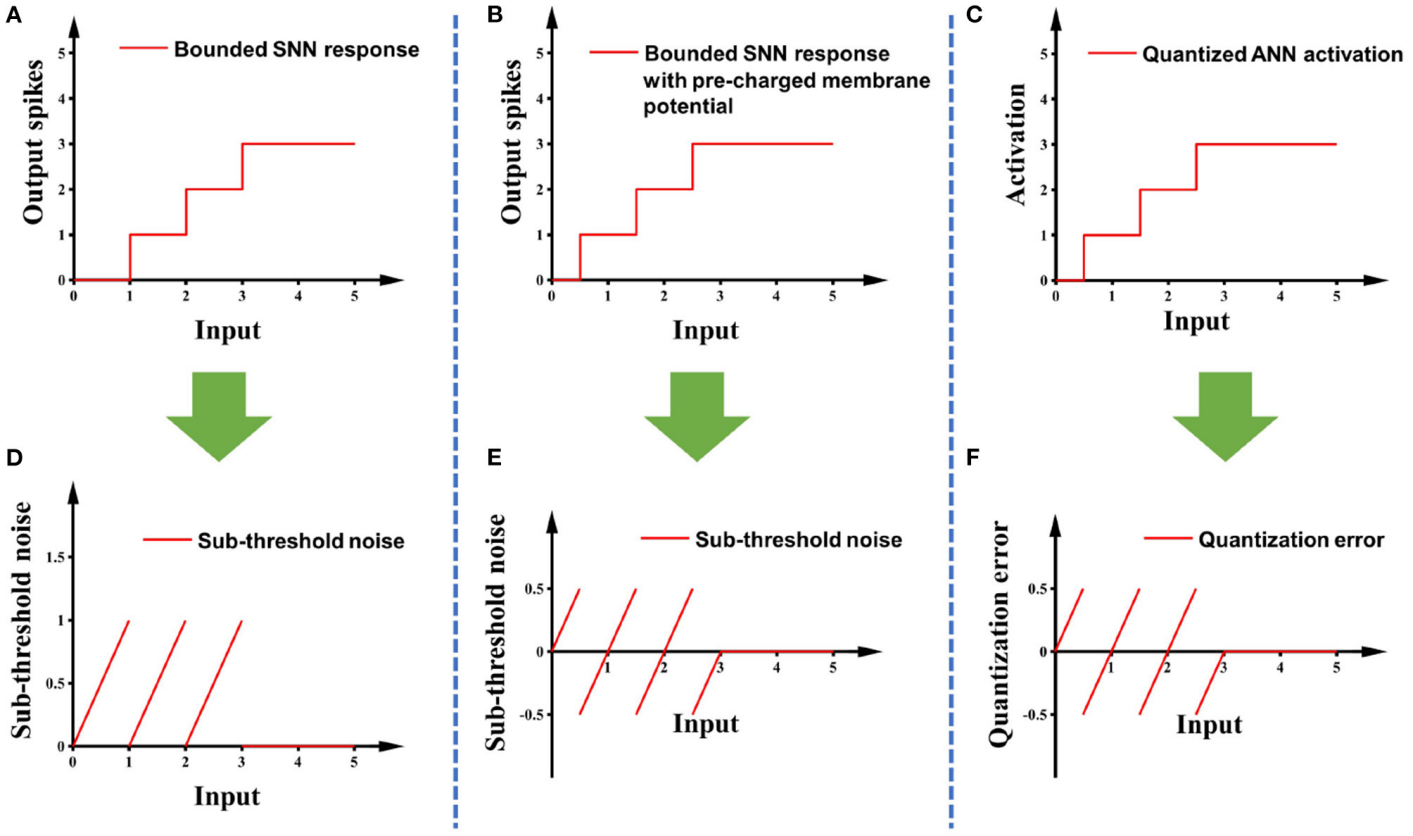
**(A)** The response curve of the modified IF model with the maximum spike count bound of 3; **(B)** adding pre-charged membrane potential to **(A)**; **(C)** the response curve of a 2-bit quantified ANN; **(D,E)** are the sub-threshold noise of **(A,B)**, respectively, compared with clipped ReLU; **(F)** is the quantization error of **(C)** relative to clipped ReLU.

Sub-threshold noise is the residual membrane potential of spiking neurons after simulation which may cause the output of spiking neurons to be lower than the expected value (Diehl et al., 2015). The solutions proposed in previous research are either bias shift (Deng and Gu, 2021) or pre-charged membrane potential (Hwang et al., 2021). We choose to use pre-charged membrane potential to reduce the amplitude of this noise. After applying the pre-charged membrane potential, the sub-threshold noise shares the same amplitude and patterns as the quantization error in the ANN, so the sub-threshold noise is canceled out. Detailed illustrations are in Figure 4.

#### 4.4.2. Handling Occasional Noise

To avoid the negative impact of occasional noise, it is necessary to identify its pattern of occurrence. Occasional noise only occurs when:

- The membrane potential of a spiking neuron after the simulation is negative, but at least one spike has been generated during the simulation. This means that more spikes were generated than there should have been.
- The membrane potential of a spiking neuron after the simulation is higher than the threshold, which means that the generated spike count is lower than expected.

The spiking neurons that fit either of these two situations are considered to suffer the impact of occasional noise. Here we provide a feasible solution to mitigate the occasional noise. The main feature of our proposed method is that it can compensate for the occasional noise during the simulation instead of after the simulation. In other words, the event-based nature of SNNs is maintained.

To handle the first situation, we add a mechanism for generating negative spikes in spiking neurons to compensate for the incorrectly emitted positive spikes: A negative spike will be generated when the membrane potential is smaller than zero and the total spike count generated by this neuron is greater than zero. These two prerequisites correspond to the two features listed in the first situation. After a negative spike is generated, the membrane potential will increase by a value equal to its threshold, which is opposite to the reset mechanism of the positive spike. A maximum spike count is set to mimic the maximum quantization value in activation-quantized ANNs. The pseudo-code of this spiking model is given in Algorithm 1. There are two parameters in this spiking model that need to be defined, the threshold of the spiking neurons *th* and the Maximum spike count limitation *Z*_*max*. How these parameters can be determined is illustrated in Section 4.6. The simulation time of the SNN is extended correspondingly to enable these newly-generated spikes to propagate to deep layers.

**Algorithm 1:** The spiking neuronal model.

**Input**: Spiking neuron's input *x*
**Parameter**: Spiking neuron's voltage *u*, Current time-step *t*, Time window *T*, Threshold *th*, Generated spike count *Z*, Maximum spike count *Z*_*max*, Heaviside step function Θ
**Output**: Generated spike *z*
1: LET *u*_−1_ = 0, *z*_−1_ = 0, *Z*_−1_ = 0
2: **for** *t* ∈ [0, *T*] **do**
3: *u*_*t*_ = *u*_*t*−1_ − *z*_*t*−1_*th* + *x*
4: **if** (*u*_*t*_ ≥ *th*)*and*(*Z*_*t*−1_ < *Z*_*max*) **then**
5: *z*_*t*_ = Θ(*u*_*t*_ − *th*)
6: **else if** (*u*_*t*_ ≤ 0)*and*(*Z*_*t*−1_ > 0) **then**
7: *z*_*t*_ = −Θ(−*u*_*t*_)
8: **else**
9: *z*_*t*_ = 0
10: **end if**
11: *Z*_*t*_ = *Z*_*t*−1_ + *z*_*t*_
12: **end for**

**Algorithm 2:** Event-based max pooling.

**Input**: The accumulated spike counts of spiking neurons before the max pooling layer *Z*
**Parameter**: Max pooling *max*_*pooling*, the output of max pooling *M*, Current time-step *t*, Time window *T*
**Output**: Generated spike *z*
1: LET *z*_−1_ = 0,*M*_−1_ = 0
2: **for** *t* ∈ [0, *T*] **do**
3: *M*_*t*_ = *max*_*pooling*(*Z*_*t*_)
4: *z*_*t*_ = *M*_*t*_ − *M*_*t*−1_
5: **end for**

In order to deal with the second situation, we simply extend the simulation time to allow the spike to emit.

### 4.5. Event-Based Max Pooling

Max pooling is problematic for rate-coded SNNs due to their fundamentally different ways of representing information. In ANNs, information is represented by activation values, while in rate-coded SNNs, information is represented by the number of accumulated spikes over time. In each time step, only a limited amount of information is carried by a spike, so simply conducting max pooling on a spike for each time step will introduce considerable accuracy loss. Using winner-take-all mechanisms to model max pooling is more biologically plausible, yet sometimes the winner may not be the one with the maximum activation value.

Here we discuss this problem and provide a practical approach to implement max pooling in SNNs. Basically, max pooling in ANNs is picking the maximum value from a series of values in the previous layer. These values are represented as spike counts if the SNNs are rate-coded, so the output of the max pooling should be the maximum value of these spike counts. Note that spike counts need to be recorded after all spikes are generated, to prevent the miscounting of spikes. For example, when calculating the max pooling in a layer of a SNN, assuming the time window of this SNN is *T*, and the totally accumulated spike counts recorded at time *T* is *Z*_*T*_, then the max pooling output *M*_*T*_ would be


(3) $$\begin{array}{l} M_{T}=max\text{\_}pooling\left(Z_{T}\right) \end{array}$$

where *max*_*pooling* is the max pooling operation. The limitation of this method is obvious. The max pooling output can only be obtained at time *T* after all spikes have been generated in the previous layer, which violates the event-based nature of SNNs.

To protect the event-based nature of SNNs, we add some modifications to the method above: for each time step *t*, the max pooling on spike counts *Z*_*t*_ are recorded as


(4) $$\begin{array}{l} M_{t}=max\text{\_}pooling\left(Z_{t}\right) \end{array}$$

The output of max pooling at time *t* is defined as


(5) $$\begin{array}{l} z_{t}=M_{t}-M_{t-1} \end{array}$$

Using this approach, a max pooling output spike *z*_*t*_ is generated only when *M*_*t*_ changes, which keeps the event-based nature of SNNs. All generated spikes $\overset{T}{\underset{t=1}{\sum }}z_{t}$ during the simulation will be the target max pooling output *M*_*T*_, which is explained below:

According to Equation (5), the accumulated spike output of max pooling $\overset{T}{\underset{t=1}{\sum }}z_{t}$ would be


$$\begin{array}{l} \overset{T}{\underset{t=1}{\sum }}z_{t}=\overset{T}{\underset{t=1}{\sum }}\left(M_{t}-M_{t-1}\right)\\ =\left(M_{T}-M_{T-1}\right)+\left(M_{T-1}-M_{T-2}\right)\cdot \cdot \cdot \left(M_{0}-M_{-1}\right)\\ =M_{T}+\left(-M_{T-1}+M_{T-1}\right)+\left(-M_{T-2}+M_{T-2}\right)\cdot \cdot \cdot \\ \left(-M_{0}+M_{0}\right)-M_{-1}\\ =M_{T}-M_{-1} \end{array}$$

We can see that all intermediate terms are canceled out, and the last term *M*_−1_ is 0, so this equation becomes


(6) $$\begin{array}{l} \overset{T}{\underset{t=1}{\sum }}z_{t}=M_{T} \end{array}$$

The result equals that in Equation (3) from conducting max pooling on spike counts.

Access to the spike count and the calculation of the difference are uncomplicated and can be implemented in PyNN (Davison et al., 2009) and PyTorch-based SNN simulation platforms such as snnTorch and SpikingJelly, which offer compatibility with our method. Also, the nature of event-based computing in SNNs is preserved in our proposed method.

### 4.6. Quantization Meets ANN-to-SNN Conversion

This section provides detailed equations relating to the general ANN-to-SNN conversion and the proposed Quant-ANN-to-SNN conversion. At the end of this section, we show that Quant-ANN-to-SNN conversion is a special form of the general ANN-to-SNN conversion.

#### 4.6.1. ANN-to-SNN Conversion

In an ANN, the information processing in the artificial neurons in layer *l* can be modeled as


(7) $$\begin{array}{l} y^{l}=a\left(W^{l}y^{l-1}+B^{l}\right) \end{array}$$

where *a*(·) is the ReLU activation function, ***W***^*l*^ and ***B***^*l*^ denote the weight and the bias in layer *l*, ***y***^*l*^ is the output of layer *l*, and ***y***^*l*−1^ is the output of layer *l* − 1 (which is also the input to layer *l*).

Meanwhile, the integrate-and-fire model used in an SNN is defined as


(8) $$\begin{array}{l} u_{t}^{l}=u_{t-1}^{l}+\tilde{W^{l}}z_{t}^{l-1}{\mathrm{th}}^{l-1}+\tilde{B^{l}}-z_{t-1}^{l}{\mathrm{th}}^{l} \end{array}$$

(9) $$\begin{array}{l} z_{t}^{l}=\Theta \left(u_{t}^{l}-{\mathrm{th}}^{l}\right) \end{array}$$

where $u_{t}^{l}$ and $u_{t-1}^{l}$ are the membrane potential of spiking neurons in layer *l* at time *t* and *t* − 1 respectively, $\tilde{W^{l}}$ is the weight and $\tilde{B^{l}}$ is the bias. Θ denotes the Heaviside step function. *th*^*l*^ is the threshold in layer *l*. $z_{t}^{l}$ is the output spike in this layer at time *t*. Note that the reset mechanism in this spiking neuronal model is the reset-by-subtraction rather than the reset-to-zero.

When conducting ANN-to-SNN conversion based on the data-based normalization, the SNN parameters $\tilde{W^{l}}$, $\tilde{B^{l}}$ and *th*^*l*^ are calculated by


(10) $$\begin{array}{l} \tilde{W^{l}}=\frac{\lambda^{l-1}W^{l}}{\lambda^{l}} \end{array}$$

(11) $$\begin{array}{l} \tilde{B^{l}}=\frac{B^{l}}{\lambda^{l}} \end{array}$$

(12) $$\begin{array}{l} {\mathrm{th}}^{l}=1 \end{array}$$

where λ^*l*^ and λ^*l*−1^ are the maximum ANN activation value in layer *l* and the previous layer *l* − 1.

#### 4.6.2. Quant-ANN-to-SNN Conversion

In an activation-quantized ANN, the activation function is defined by


(13) $$\begin{array}{l} y^{l}=s^{l}\times \left\lfloor clip\left(\frac{W^{l}\cdot y^{l-1}+B^{l}}{s^{l}},0,2^{B}-1\right)\right\rceil , \end{array}$$

where *s*^*l*^ is the quantization step size in layer *l*, and it is the only parameter that is not predefined but is learned during quantization training. *b* is the activation bit precision so 2^*b*^ − 1 is the maximum quantization value in this ANN. *clip*(*a, b, c*) clips *a* with the value below *b* set to *b* and the value above *c* set to *c*. ⌊*a*⌉ rounds *a* to the nearest integer. This process comprising scaling, clipping, rounding and re-scaling is applying a fake quantization to ANN activation.

After conducting Quant-ANN-to-SNN conversion, the applied spiking neuronal model is described in Algorithm 1, or defined by the equations below:


(14) $$\begin{array}{l} u_{t}^{l}=u_{t-1}^{l}+\tilde{W^{l}}z_{t}^{l-1}{\mathrm{th}}^{l-1}+\tilde{B^{l}}-z_{t-1}^{l}{\mathrm{th}}^{l} \end{array}$$

(15) $$\begin{array}{l} z_{t}^{l}=\Theta \left(u_{t}^{l}-{\mathrm{th}}^{l}\right)\Theta \left(Z\text{\_}max-Z_{t-1}^{l}\right)-\Theta \left(-u_{t}^{l}\right)\Theta \left(Z_{t-1}^{l}\right) \end{array}$$

(16) $$\begin{array}{l} Z_{t}^{l}=Z_{t-1}^{l}+z_{t}^{l} \end{array}$$

The meaning of these items is in Algorithm 1. $\Theta \left(u_{t}^{l}-{\mathrm{th}}^{l}\right)\Theta \left(Z\text{\_}max-Z_{t-1}^{l}\right)$ determines whether a spike is generated, where $\Theta \left(Z\text{\_}max-Z_{t-1}^{l}\right)$ prevents emitting more spike than *Z*_*max*. $-\Theta \left(-u_{t}^{l}\right)\Theta \left(Z_{t-1}^{l}\right)$ identify the occasional noise and compensate it by generating a negative spike. The SNN parameters are calculated by


(17) $$\begin{array}{l} \tilde{W^{l}}={\mathrm{th}}^{l-1}W^{l} \end{array}$$

(18) $$\begin{array}{l} \tilde{B^{l}}=B^{l} \end{array}$$

(19) $$\begin{array}{l} {\mathrm{th}}^{l}=\left(2^{B}-1\right)s^{l} \end{array}$$

(20) $$\begin{array}{l} Z\text{\_}max=2^{B}-1 \end{array}$$

By using the spiking neuronal model described in Equations (14–16) and normalizing SNN parameters by Equations (17–20), a lossless Quant-ANN-to-SNN conversion can be achieved.

#### 4.6.3. Connection Between ANN-to-SNN Conversion and Quant-ANN-to-SNN Conversion

The connection between ANN-to-SNN conversion and Quant-ANN-to-SNN conversion is illustrated below. Equations (17–19) are significantly different from the data-based normalization (Equations 10–12). However, one characteristic of spiking neural networks is that the function of an SNN will be unchanged after scaling weights, bias, and spiking thresholds simultaneously. If we scale these parameters by 1/(2^*b*^ − 1)*s*^*l*^ simultaneously, these equations become


(21) $$\begin{array}{l} \tilde{W^{l}}=\frac{\left(2^{B}-1\right)s^{l-1}W^{l}}{\left(2^{B}-1\right)s^{l}} \end{array}$$

(22) $$\begin{array}{l} \tilde{B^{l}}=\frac{B^{l}}{\left(2^{B}-1\right)s^{l}} \end{array}$$

(23) $$\begin{array}{l} {\mathrm{th}}^{l}=1 \end{array}$$

We can see that these equations become more similar to the data-based normalization (Equations 10–12). For instance, (2^*b*^ − 1)*s*^*l*^ in Equation (21) corresponds to λ^*l*^ in Equation 10, and they are both the maximum output value in an ANN layer. This shows the internal correspondence of our method to traditional ANN-to-SNN conversion techniques.

## 5. Experiments

### 5.1. Experimental Setup

We conduct quantization training based on pre-trained full precision VGG-16 and ResNet models. The networks were trained by stochastic gradient descent with the loss function of cross-entropy and the exponential decay scheduler. Detailed hyper-parameters are in Table 2. We chose 2-bit activation precision in all hidden layers for CIFAR-10 and ImageNet in quantization training to render the best SNN latency. Notably, the output layer is 3-bit, and the reasons for this choice are discussed in the following sections. Both ANN quantization training and SNN implementation are carried out with PyTorch.

**Table 2:** Hyper-parameters of ANN quantization training.

Learning rate	0.01
Momentum	0.9
Weight decay	0.0005
Epoch	40
Batch size	32
Other technique	Data augmentation

In SNN simulation, the network input is analog-coded (Rueckauer et al., 2017) and the time resolution is 1ms. The adopted neuronal model was described in Algorithm 1. The maximum spike count is limited to 2^*b*^ − 1 in hidden layers and 2^*b*+1^ − 1 in the output layer, which corresponds to the maximum quantization states during quantization training. *b* is the activation bit precision during ANN quantization training and is chosen as 2. The weight ${\tilde{W}}^{l}$, the bias ${\tilde{B}}^{l}$, the threshold *th*^*l*^ and the maximum spike count limitation *Z*_*max* in layer *l* are determined by


(24) $$\begin{array}{l} \tilde{W^{1}}={\mathrm{th}}^{l-1}W^{l} \end{array}$$

(25) $$\begin{array}{l} \tilde{B^{1}}=B^{l} \end{array}$$

(26) $$\begin{array}{l} {\mathrm{th}}^{l}=\left(2^{B}-1\right)s^{l} \end{array}$$

(27) $$\begin{array}{l} Z\text{\_}max=2^{B}-1 \end{array}$$

where ***W***^*l*^, ***B***^*l*^ and *s*^*l*^ are the weight, the bias and the quantization step size in layer *l* which are learned during ANN quantization training (Esser et al., 2019). The membrane potential of the spiking neurons in all layers is pre-charged by 0.5*th* at the first time step to eliminate systematic errors relative to the quantized ANNs (Hwang et al., 2021).

Bias in the SNNs is modeled by constant current injection into the spiking neurons (Rueckauer et al., 2017). After time step 2^*b*^ − 1, both the bias and the network input are shut down, so only the spikes caused by occasional noise can be passed through the network.

### 5.2. Benchmark Results

The following benchmark results are on ResNet models. More results on VGG-16 models are in Section 5.6.

After converting quantized ANNs to SNNs, an accuracy of 93.14% is achieved within 4 time steps on CIFAR-10 and an accuracy of 70.18% is reached within 8 time steps on ImageNet. Compared with previous work on ANN-to-SNN conversion, the inference latency of the SNNs is shortened significantly while retaining competitive accuracy as shown in Table 3.

**Table 3:** Benchmarking SNNs built by ANN-to-SNN conversion on CIFAR-10 and on ImageNet.

**Method**	**Dataset**	**Architecture**	**Acc(ANN)(%)**	**Acc(SNN)(%)**	**Latency (ms)**
RNL+RIL (Ding et al., 2021)	CIFAR-10	ResNet-18	93.06	91.96	64
RNL+RIL (Ding et al., 2021)	CIFAR-10	VGG-16	92.82	91.15	64
TCL (Ho and Chang, 2020)	CIFAR-10	ResNet-20	91.58	91.22	35
TCL (Ho and Chang, 2020)	CIFAR-10	VGG-16	93.25	92.6	20
QCFS (Bu et al., 2021)	CIFAR-10	ResNet-20	91.77	91.62	16
QCFS (Bu et al., 2021)	CIFAR-10	ResNet-18	**96.04**	94.82	8
QCFS (Bu et al., 2021)	CIFAR-10	VGG-16	95.52	**94.95**	8
TS (Deng and Gu, 2021)	CIFAR-10	ResNet-20	92.32	92.41	16
TS (Deng and Gu, 2021)	CIFAR-10	VGG-16	92.09	92.29	16
**QFFS (This work)**	CIFAR-10	ResNet-18	93.12	93.14	**4**
**QFFS (This work)**	CIFAR-10	VGG-16	92.44	92.64	**4**
Spike-Norm (Sengupta et al., 2019)	ImageNet	VGG-16	70.52	69.96	2500
Spike-Norm (Sengupta et al., 2019)	ImageNet	ResNet-34	70.69	65.47	2000
RMP (Han et al., 2020)	ImageNet	VGG-16	73.49	73.09	4096
RMP (Han et al., 2020)	ImageNet	ResNet-34	70.64	69.89	4096
TCL (Ho and Chang, 2020)	ImageNet	VGG-16	73.22	70.75	30
TCL (Ho and Chang, 2020)	ImageNet	ResNet-34	70.85	70.37	250
QCFS (Bu et al., 2021)	ImageNet	VGG-16	74.29	72.85	64
QCFS (Bu et al., 2021)	ImageNet	ResNet-34	**74.32**	72.35	64
TS (Deng and Gu, 2021)	ImageNet	VGG-16	72.4	70.97	64
**QFFS (This work)**	ImageNet	ResNet-50	70.15	70.18	8
**QFFS (This work)**	ImageNet	VGG-16	69.88	69.69	8
**QFFS with analog output (This work)**	ImageNet	ResNet-50	72.81(74.07)	72.91(**74.36**)	5(10)
**QFFS with analog output(This work)**	ImageNet	VGG-16	71.88(73.08)	72.10(73.10)	**4**(8)

Note that in fast SNN research, sometimes the SNN accuracy will be higher than the ANN accuracy. This phenomenon usually appears when an SNN is converted from an ANN whose activation function is clipped ReLU, or when the dataset is less challenging such as CIFAR-10. This has been reported in several studies but an adequate explanation is still lacking (Deng and Gu, 2021; Ding et al., 2021). Our research also shows higher SNN accuracy than that in the ANN in some experimental settings—the extreme case is that the accuracy of a SNN is about 0.3% higher than its ANN accuracy, as shown in Table 3. The reason may be that the applied ANN activation function contains a clipped point such as in the clipped ReLU. Another potential reason may be the difference in information processing mechanisms between ANNs and SNNs. ANNs calculate the activation function by multiplication, while SNNs calculate outputs using their integrate-and-fire mechanism. Thus, even if a perfect ANN-to-SNN conversion is conducted, a value in the ANN may be represented as a slightly different value in the SNN.

**Figure 5:**
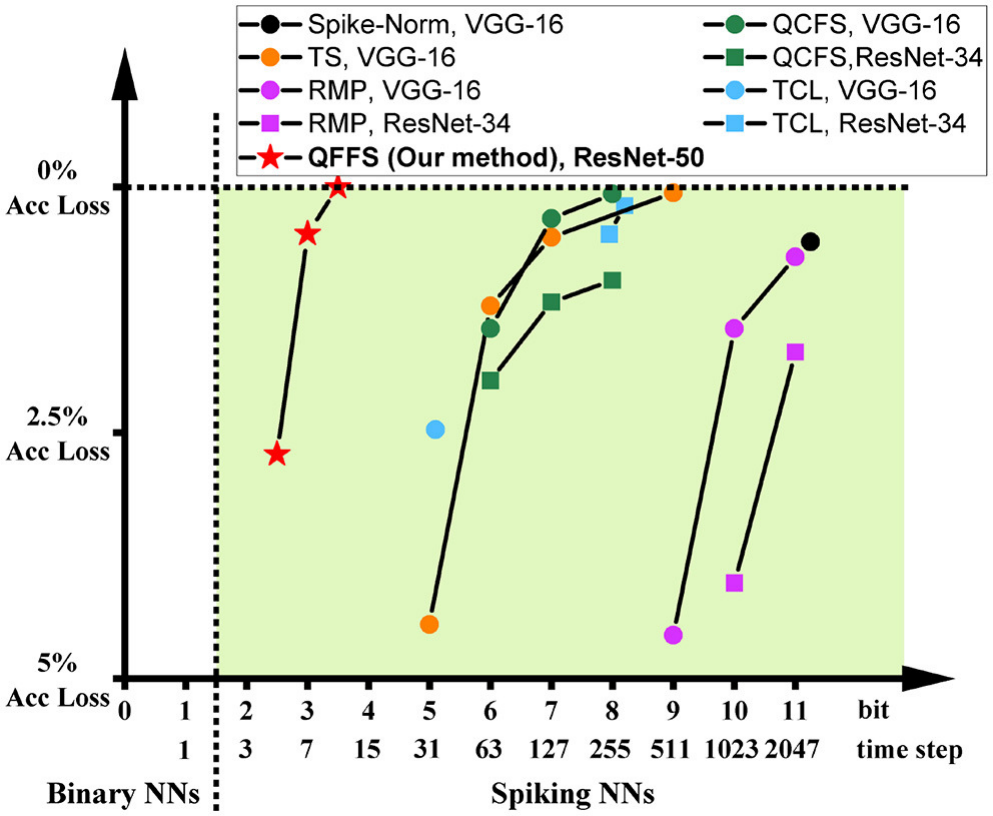
The relationship between ANN-to-SNN conversion loss and SNN inference latency on ImageNet.

We also benchmark the required time steps to achieve lossless ANN-to-SNN conversion on ImageNet; (see Figure 5). The y-axis represents the accuracy gap to the baseline ANN accuracy before the conversion. The horizontal axis is the required number of time steps of the SNNs, which is converted into the equivalent bit resolution at the bottom. What stands out in this figure is that our proposed quantization framework for fast SNNs only needs 13 time steps—about 4 bits of information—to reach lossless accuracy, while other methods need at least 500 time steps, or 9 bits of information, to achieve lossless accuracy. This highlights the merit of applying information compression techniques to SNNs and the effectiveness of the proposed Quant-ANN-to-SNN conversion paradigm.

### 5.3. Bit Precision During Quantization Training

**Figure 6:**
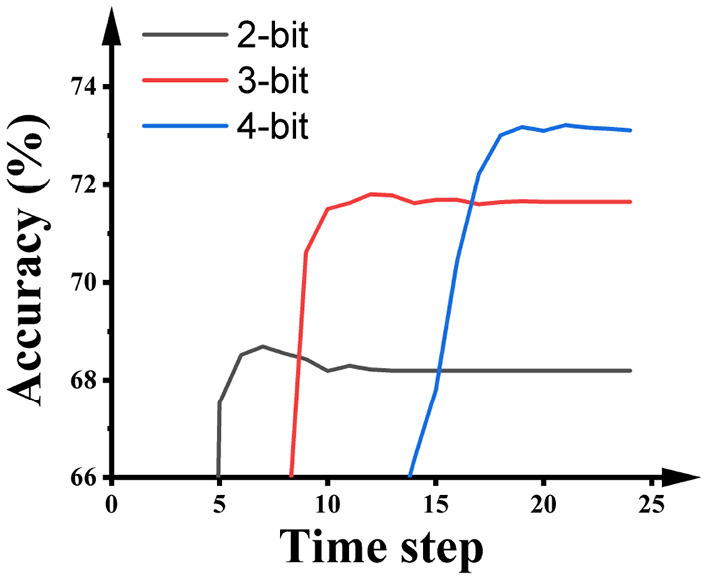
The impact of the activation precision during the quantization training of ResNet-50 on the performance of SNNs on ImageNet.

Figure 6 illustrates the impact of the activation precision during quantization training on the accuracy and latency of SNNs on ImageNet. As shown in the figure, higher activation precision during quantization training will offer higher accuracy in the SNNs, while the number of time steps required by the SNNs is extended. Hence, there is an accuracy-latency trade-off inside the Quant-ANN-to-SNN conversion technique.

**Figure 7:**
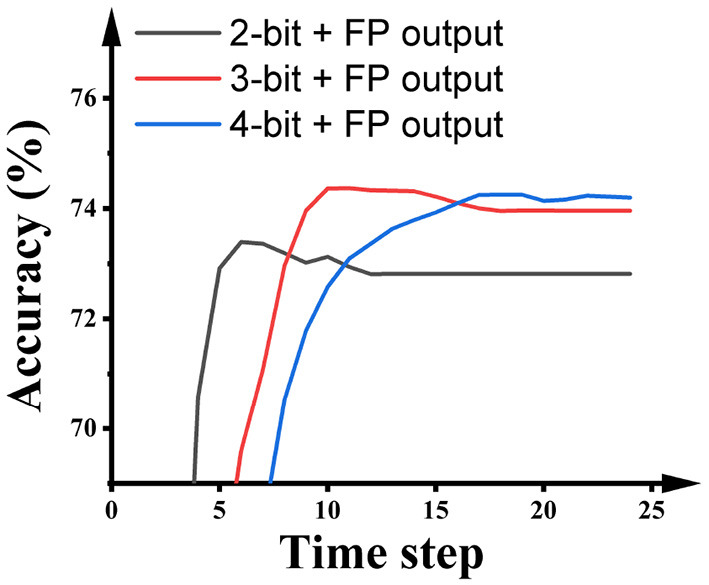
The impact of the activation precision in hidden layers during the quantization training of ResNet-50 on the performance of SNNs on ImageNet.

Some SNN research keeps the output layer as floating-point and suggests that this promotes inference accuracy. For a fair comparison with this kind of research, we also report the performance of SNNs built using this paradigm. As shown in Figure 7, using full precision in the output layer during ANN quantization training obviously improves the inference accuracy and latency of the SNNs. Particularly, the 2-bit SNN reaches 72.91% in 5 time steps, and the 3-bit SNN reaches 74.36% in 10 time steps.

### 5.4. Bit Precision in the Output Layer

The results in the previous section show that the network accuracy is very sensitive to the bit precision in the output layer. For instance, changing the output layer from 2-bit to floating-point gives a 4% accuracy improvement on ImageNet. In this section, we provide more fine-grained results on the bit precision in the output layer. Also, we discuss improving inference accuracy without sacrificing biological plausibility, in particular without using analog neurons in the output layer of the SNNs.

We mentioned that the SNN converted from a 2-bit ANN needs about 7 time steps to reach the highest accuracy as shown in Figure 6, which means that most of the information has been transmitted to the output layer in 7 time steps. Seven time steps can represent 3 bits of information from a rate-coded spiking neuron in theory, while the bit precision of the output layer is only 2 bits. This gap motivates us to further utilize the information representation ability of spiking neurons by adjusting the precision of the output layer.

**Figure 8:**
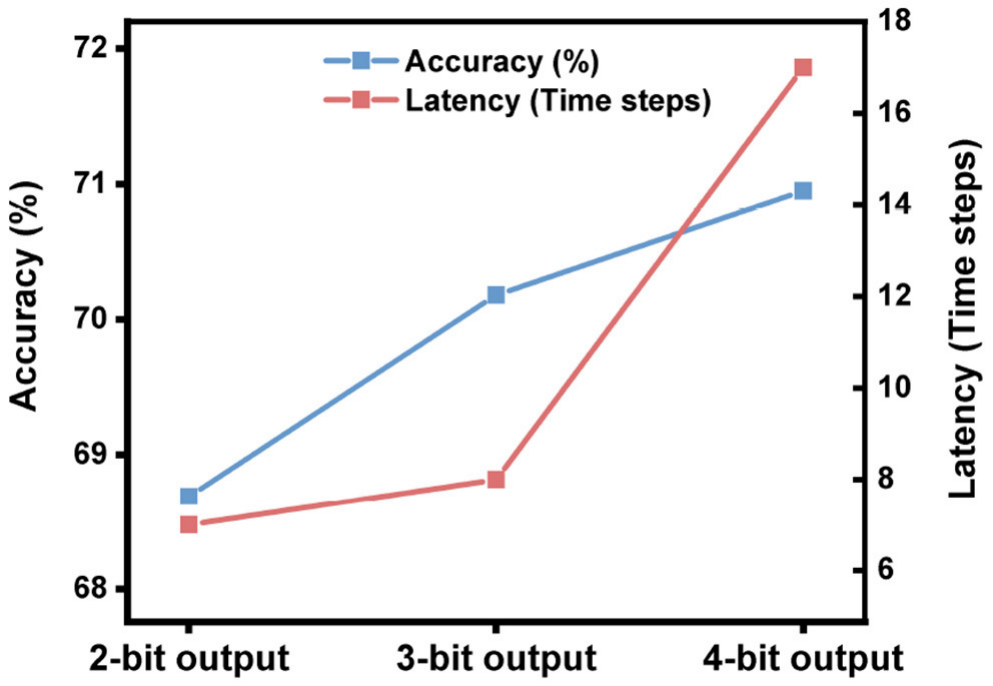
The impact of the activation precision in the output layer during ANN quantization on the performance of SNNs on ImageNet. The network architecture is ResNet-50.

We keep the bit precision in all hidden layers as 2-bit, and adjust the bit precision in the output layer during quantization training. As shown in Figure 8, higher precision in the output layer brings higher accuracy but longer latency. What stands out in this figure is the case with 3-bit bit precision: its accuracy improves 1.49% while its latency is only extended by 1 time step compared with the case with 2-bit precision. Meanwhile, its latency is 2 times shorter than the case with 4-bit precision. With that in mind, we choose 2-bit in all hidden layers and 3-bit in the output layer during quantization training to build high-accuracy, low-latency SNNs.

### 5.5. Ablation Studies

We decompose the effect of our methods using an ablation study as shown in Table 4. The default setting is the SNN converted from a 2-bit quantized ANN. After applying the mechanisms of generating negative spikes and extending the simulation time to let the newly generated spikes propagate, the accuracy reaches 67.29%. Using max pooling in the network increases the SNN accuracy to 68.69% compared with using average pooling. Another 1.49% accuracy improvement comes from using 3-bit quantization in the output layer.

**Table 4:** Ablation studies on ImageNet.

**Index**	**Setting**	**Accuracy(%)**
①	Default	0.01
②	① + Negative spike	10.64
③	② + Simulation time step extension	67.29
④	③ + Max pooling	68.69
⑤	④ + More bits in the output layer	70.18

### 5.6. Results on VGG-16

**Figure 9:**
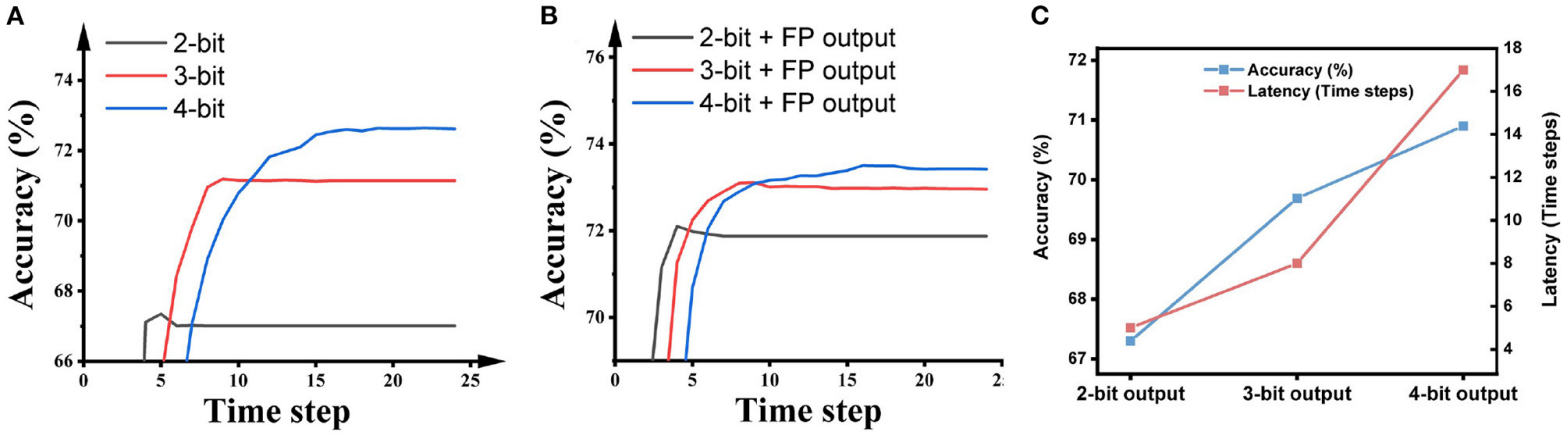
The impact of the bit precision in **(A)** all layers, **(B)** all hidden layers, and **(C)** the output layer on SNN accuracy and latency. The output precision in **(B)** is floating-point; the precision of hidden layers in **(C)** is 2 bits. The dataset is ImageNet and the network architecture is VGG-16.

The SNNs applying the VGG-16 network architecture achieved an accuracy of 92.62% in 4 time steps on CIFAR-10 and an accuracy of 69.69% in 8 time steps on ImageNet. The results on the impact of bit precision, the bit precision in the hidden layers, and the bit precision in the output layer are shown in Figure 9.

## 6. Discussion and Further Improvements Based on QFFS

By establishing a bridge from ANN quantization precision to SNN inference latency, we achieved state-of-the-art inference latency in SNNs. This demonstration significantly improves the performance of rate-coded SNNs, and should facilitate future SNN implementations on edge devices for ultra-fast, event-based computing. SNNs encoded by temporal coding may also benefit from this research as, in these encoding schemes, the amount of information to be encoded is crucial as well.

This study offers a fresh perspective on how to generate more rapid progress on SNNs: in our proposed quantization framework for fast SNNs, the first step is selecting one effective technique (instead of developing an SNN algorithm from scratch). The remaining three steps in QFFS are making the knowledge transmission from ANNs to SNNs smoother. Considering the prosperity in current ANN research, we believe this research concept will continue to work in the near future.

This research provides a novel method to achieve lossless ANN-to-SNN conversion within several time steps. Furthermore, we have built a framework to facilitate future improvements in fast SNN research: The accuracy can be increased by improving the first step in QFFS, which is quantizing the ANN activation as shown in Figure 2. The method we chose to conduct quantization training is LSQ. However, quantization training techniques are developing rapidly, and there are other effective methods being proposed after LSQ. We suggest that the progress in ANN quantization techniques can promote accuracy improvements in fast SNN research through the bridge built by our proposed framework.

The four identified types of noise are the main cause of the degradation in accuracy and the extension in latency. In this research, the noise suppression measures are considered only after the ANN-to-SNN conversion. An alternative method is to suppress noise before conducting the ANN-to-SNN conversion. For example, it may be helpful if some constraints can be added during quantization training to make SNNs robust to occasional noise. In this case, the noise could be suppressed more effectively, and the latency of SNNs may be improved.

Furthermore, occasional noise is suppressed by modifying spiking neuronal models (the third step in Figure 2). It is worthwhile to study how to use more biologically plausible mechanisms to suppress occasional noise in the future, thereby further improving the proposed QFFS.

## Data Availability Statement

Publicly available datasets were analyzed in this study. This data can be found here: https://image-net.org/, and on the website of CIFAR-10: https://www.cs.toronto.edu/~kriz/cifar.html.

## Author Contributions

CL developed the methods, under the supervision of SF. LM provided GPU resources and funding during CL's visit to BAAI. All authors contributed to the article and agreed to the submission.

## Funding

This research has received funding from the European Union's Horizon 2020 Framework Programme for Research and Innovation under the Specific Grant Agreement No. 945539 (Human Brain Project SGA3). This work was supported in part by National Key R&D Program of China (Nos. 2020AAA0105200, 2021ZD0109802).

## Conflict of Interest

The authors declare that the research was conducted in the absence of any commercial or financial relationships that could be construed as a potential conflict of interest.

## Publisher's Note

All claims expressed in this article are solely those of the authors and do not necessarily represent those of their affiliated organizations, or those of the publisher, the editors and the reviewers. Any product that may be evaluated in this article, or claim that may be made by its manufacturer, is not guaranteed or endorsed by the publisher.
